# Effect of emodin on the cariogenic properties of *Streptococcus mutans* and the development of caries in rats

**DOI:** 10.3892/etm.2014.1857

**Published:** 2014-07-21

**Authors:** JING-SHU XU, YUN CUI, XIAN-MIN LIAO, XIAO-BIN TAN, XUE CAO

**Affiliations:** Department of Oral Medicine, Kunhua Hospital, The First People’s Hospital of Yunnan Province, Kunming, Yunnan 650032, P.R. China

**Keywords:** caries, *Streptococcus mutans*, glucosyltransferase, emodin

## Abstract

Emodin is an active herbal component traditionally used in East Asian countries for treating a variety of diseases. The present study investigated the effects of emodin on specific virulence factors of *Streptococcus mutans* (*S. mutans*) *in vitro* and on caries development *in vivo*. The growth and acid production of *S. mutans* were significantly inhibited by emodin (0.5–2 mg/ml). Emodin also significantly suppressed the synthesis of insoluble glucans by *S. mutans*. Furthermore, the topical application of emodin reduced the incidence and severity of carious lesions in rats. These results suggest that the natural compound emodin may be a novel pharmacological agent for the prevention and treatment of dental caries.

## Introduction

Dental caries is an infectious oral disease prevalent across the world and it is associated with various pathogenic microorganisms. *Streptococcus mutans* (*S. mutans*) is considered a crucial pathogen in the pathogenesis of dental caries (1). It is involved in the development and establishment of cariogenic biofilms. The major factors responsible for the cariogenicity of this pathogen include its ability to produce glucosyltransferases (Gtfs), synthesize insoluble glucans, generate acids and survive at low pH values (2–5). Therefore, a previous study hypothesized that disrupting the ability of *S. mutans* to form acids and glucans may be an effective therapeutic approach for the treatment of dental caries (6).

Despite advances in the development of anti-caries chemotherapy, conventional therapeutic strategies are often unable to control the progression of dental caries. It has been reported that the use of natural products is one of the most successful strategies for the discovery of new medicines (7). The herbaceous plants *Polygonum* (Polygonaceae), *Rhamnus* (Rhamnaceae) and *Senna* (Fabaceae) have been successfully used as traditional medicines in East Asian countries (8,9). These herbs have demonstrated various pharmacological effects; for example, Polygonaceae have the ability to control dental diseases (10). Furthermore, a previous study revealed that extracts from Polygonaceae roots are able to inhibit the production of acid by *S. mutans*, suggesting that they may be useful for the treatment of dental caries (11). Emodin (1,3,8-trihydroxy-6-methylanthraquinone) is a major active component commonly present in these herbaceous plants. It has been reported that emodin exhibits a wide range of biological activities including antibacterial, anticancer, anti-inflammatory, anti-diabetic and anti-oxidative activities (12–14). Considering that emodin is the main component of Polygonaceae, it may be meaningful to evaluate the biological effects of emodin on dental caries. The aim of the present study was to examine the effects of emodin on the growth, acid production and insoluble glucan synthesis of *S. mutans in vitro*, and caries development *in vivo*.

## Materials and methods

### Materials

Emodin with purity >98% was obtained from Xi’an Tianxingjian Natural Bio-products Group (Xi’an, China). Emodin was prepared in a phosphate buffer containing 15% (v/v) ethanol. Appropriate solvent controls were included. Sodium fluoride was purchased from Sigma (St. Louis, MO, USA). *S. mutans* ATCC 25175 was provided by the Shanghai Zhi Cheng Bio-Tech Co., Ltd. (Shanghai, China). Cariogenic diet 2000 was purchased from Trophic Animal Feed High-tech Co., Ltd. (Nantong, China). All other chemicals used were of analytical grade and commercially available.

### Measurement of bacterial growth

Bacterial growth was established using previously described methods with slight modifications (15,16). Briefly, various concentrations of filter-sterilized emodin were added to 0.95 ml tryptic soy broth containing 1% glucose. *S. mutans* ATCC 25175 seed culture (0.1 ml) was inoculated into the broth medium and incubated at 37°C. The optical density of the culture was measured at 520 nm by a UV-2550 spectrophotometer (Shimadzu Corporation, Kyoto, Japan) every 1 h for 24 h.

### Measurement of the production of acid by S. mutans ATCC 25175

The acid production assay was carried out using previously described methods with slight modifications (15). Briefly, emodin was added to 0.95 ml broth containing 1% glucose and the mixture was inoculated with 0.05 ml *S. mutans* ATCC 25175 seed culture. Following incubation at 37°C for 24 h, the pH of the cultures was determined using a pH meter (pHS-3C; Shanghai REX Instrument Factory, Shanghai, China).

### Measurement of insoluble glucan synthesis by Gtfs

*S. mutans* TCC 25175 was cultured at 37°C for 24 h in tryptic soy broth. The culture supernatant was salted out with solid ammonium sulfate to 70% saturation and agitated at 4°C for 1 h. Following centrifugation at 13,500 × g for 20 min, the precipitate was dialyzed against 10 mM potassium phosphate buffer (pH 6.0). The solution of crude Gtfs was stored at −80°C until required.

The reaction mixture, consisting of 0.025 ml of the prepared solution of crude Gtfs and 0.175 ml emodin (final concentrations: 0, 0.5, 1 and 2 mg/ml) in 0.8 ml of 0.0625 M potassium phosphate buffer containing 12.5 μg/l sucrose and 0.25 μg/l sodium azide, was incubated at 37°C for 18 h. The insoluble glucan was allowed to sediment, then washed with distilled water and placed under ultrasonication for 6 sec. The absorbance was examined using a UV spectrophotometer at 520 nm against a blank.

### Gtf B activity assay

The Gtf B enzyme was obtained from the supernatant of the *S. mutans* ATCC 25175 culture and purified to almost homogeneity by hydroxyapatite column chromatography using a previously described method (17). The enzymatic activity of Gtfs was examined by incorporating [^14^C] glucose from labeled sucrose (China Isotope Corporation, Beijing, China) into the glucans. The amount of Gtf B enzyme added to every sample for all assays was equivalent to the amount required to incorporate 1 μmol of glucose during the 4 h reaction period. Purified Gtf B was mixed with different concentrations of emodin (0.5, 1 and 2 mg/ml) and incubated with [^14^C]-glucose-labeled-sucrose substrate (final concentration, 100 mM sucrose). Ethanol (final concentration, 15% v/v) was used as the control. Radiolabeled glucan was measured by scintillation counting (17,18).

### Animal study

Animal experiments were performed using previously described methods (19,20). Briefly, pathogen-free male Wistar rats (19 days of age; purchased from Kunming Medical University, Kunming, China) were infected daily for five consecutive days with a growing culture of *S. mutans* ATCC 25175. The rats, aged 25 days, were randomly divided into three groups (n=15) and their teeth were treated topically using a camel hair brush, twice daily, for five weeks as follows: i) vehicle control (15% ethanol); ii) emodin 2 mg/ml; and iii) 250 ppm fluoride. The rats were placed in individual cages and given cariogenic diet 2000 and 5% sucrose water *ad libitum*. At the end of the five-week experimental period, the rats were anesthetized and sacrificed. The lower left jaw was aseptically removed, immerged in 5.0 ml sterile saline solution and sonicated. The suspension was plated on blood agar and on Mitis Salivarius agar plus streptomycin, to respectively estimate the total number of cultivable microorganisms and *S. mutans* populations. Smooth-surface and sulcal caries and their severities (Ds, dentin exposed; Dm, 3/4 of the dentin affected; Dx, whole dentin affected) were evaluated by means of Larson’s modification of Keyes’ system (20). The caries score was determined blindly with respect to the groups. All procedures were performed in accordance with guidelines set for the use of experimental animals by the local Committee of Kunming Medical College on Animal Care and Use.

### Statistical analysis

All values are expressed as mean ± standard error of the mean. The data were analyzed using analysis of variance followed by a Tukey-Kramer multiple comparison test using SPSS 16.0 software (SPSS, Inc., Chicago, IL, USA). P<0.05 was considered to indicate a statistically significant difference.

## Results

### In vitro effect of emodin on the cariogenic properties of S. mutans

**Figure 1:**
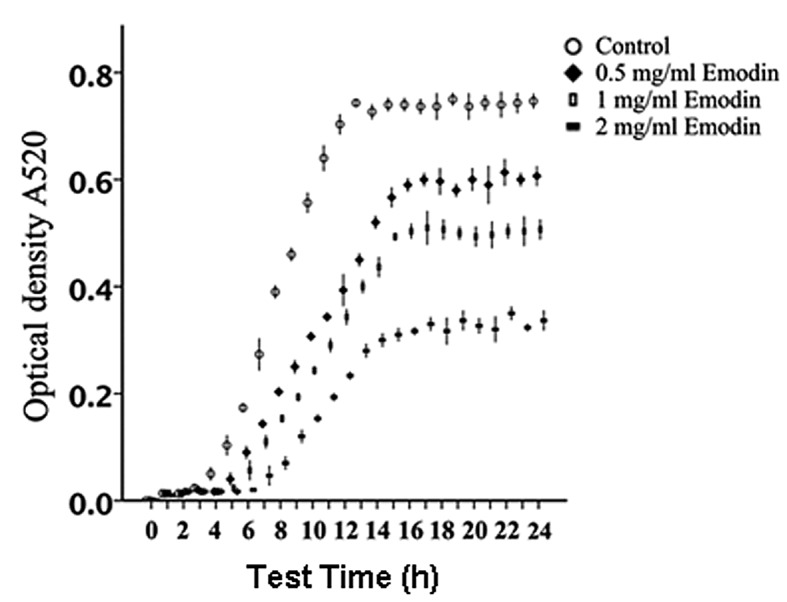
Effect of emodin on the growth of *Streptococcus mutans* ATCC 25175. The bacteria were incubated in tryptic soy broth with or without emodin at 37°C. The optical density of the cells was measured using a spectrophotometer every 1 h for 24 h. The assay was performed three times and data are expressed as mean ± standard error of the mean. ^*^P<0.05 when compared with the control treatment.

In the present study, the antibacterial effect of emodin on *S. mutans* was investigated. As shown in Fig. 1, growth of *S. mutans* ATCC 25175 was significantly reduced in the presence of emodin. This effect was revealed to be concentration dependent.

**Figure 2:**
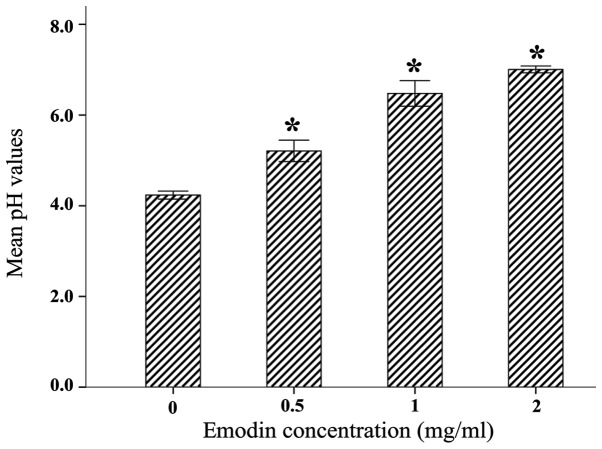
Effect of emodin on the production of acid by *Streptococcus mutans* ATCC 25175. The assay was performed three times and data are expressed as mean ± standard error of the mean. ^*^P<0.05 when compared with the control treatment.

To determine the inhibitory effect of emodin on the production of acid by *S. mutans* ATCC 25175, the cells were treated with different concentrations of emodin and the pH was measured. The addition of emodin did not change the color and pH value of cultures prior to the growth of *S. mutans* ATCC 25175 (data not shown). As shown in Fig. 2, the production of acid by *S. mutans* ATCC 25175 was significantly suppressed by emodin compared with that in the control group.

**Figure 3:**
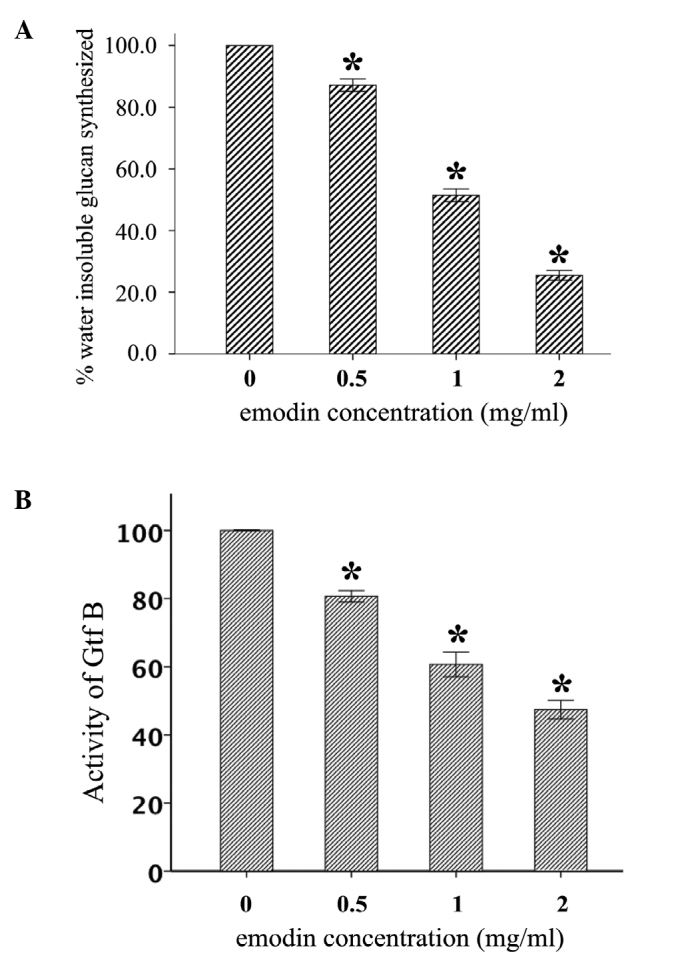
Effect of emodin on the (A) insoluble glucan synthesis and (B) glucosyltransferase (Gtf) B activity of *Streptococcus mutans* ATCC 25175. The relative amount (%) of insoluble glucan produced by various concentrations of emodin was determined as compared with the control treatment. The percentage of Gtf B activity was calculated considering the control treatment as 100% Gtf activity. The assay was performed three times and data are expressed as mean ± standard error of the mean. ^*^P<0.05 when compared with the control treatment.

Whether emodin may suppress insoluble glucan synthesis by Gtfs was also examined. As shown in Fig. 3, a significant reduction of insoluble glucan synthesis by crude Gtfs from *S. mutans* ATCC 25175 was demonstrated at concentrations >0.5 mg/ml emodin. Furthermore, emodin reduced Gtf B activity to a notable extent.

### Inhibitory effect of emodin on caries in rats

In the animal experiment, the rats remained healthy and gained weight during the five weeks of the experimental period. No significant differences in weight gain were observed among the groups (P>0.05, data not shown).

The effect of emodin on the total cultivable microorganisms, viable *S. mutans* populations and percentage of *S. mutans* recovered from the rat jaws (as calculated from the *S. mutans* and total cultivable microorganism populations) are shown in Table I. The emodin-treated group demonstrated significantly lower total microorganism counts compared with those in the vehicle control group. However, the number and percentage of *S. mutans* in the biofilms of rats treated with emodin did not differ statistically from those of the vehicle control group.

Tables II and III show the incidence and severity of smooth-surface and sulcal caries. In the present study, 250 ppm fluoride was used as a positive control. The 250 ppm fluoride treatment revealed the lowest scores for incidence and severity of smooth-surface and sulcal caries. Treatment with emodin significantly reduced the incidence of smooth-surface and sulcal caries compared with that of the vehicle control group. Furthermore, the severity scores of smooth-surface and sulcal caries were significantly lower in the group treated with emodin than in the vehicle control group.

## Discussion

Considering the high incidence rate of dental caries and its detrimental effects in the oral cavity, the development of novel strategies for its prevention and control are required. Previous studies have demonstrated that natural products are promising candidates for novel anticariogenic substances (7,21,22). The present study revealed that emodin, a natural product, interfered with key cariogenic factors of *S. mutans*, namely the synthesis of insoluble glucans and production of acid *in vitro*, and reduced the induction of caries in rats.

Emodin is a natural anthraquinone derived from the roots and rhizomes of a number of plants including *Rheum undulatum (R. undulatum)* and *Polygonum cuspidatum (P. cuspidatum)*. *P. cuspidatum* has demonstrated a broad range antibacterial effects (10). It has also been reported that the ethyl acetate fraction of *P. cuspidatum*, which is composed of polydatin, resveratrol, anthraglycoside B and emodin, is able to inhibit the glycolytic acid production and Gtf activity of *S. mutans* and *Streptococcus sobrinus* (11). The dichloromethane fraction from *R. undulatum*, composed mainly of aloe-emodin, emodin, chrysophanol and physcion, has revealed inhibitory effects on the production of glycolytic acid by *S. mutans* on biofilms (23). In the present study, emodin markedly suppressed the production of acid and the synthesis of insoluble glucan by *S. mutans* ATCC 25175. These results suggest that emodin may be responsible for the anticariogenic activity of *R. undulatum* and *P. cuspidatum*.

The synthesis of insoluble glucans is one of the most important virulent properties of *S. mutans* (24,25). Insoluble glucans promote the adhesive interaction of bacteria with the tooth surface and contribute to the formation of dental biofilms (26). Accordingly, the current study examined whether emodin may inhibit the synthesis of insoluble glucans by crude Gtfs. The results revealed that the formation of insoluble glucans was significantly suppressed by emodin. These data suggest that emodin may be a novel substance capable of modulating the activities of these important dental caries-related factors. *S. mutans* synthesizes insoluble glucans from sucrose by the action of Gtfs. There are three types of Gtfs in *S. mutans*: B, C and D. Among these, Gtfs B and C are essential virulence factors of *S. mutans* (4,27). Gtf B synthesizes primarily insoluble glucans, whereas Gtf C synthesizes a mixture of insoluble and soluble glucans. The present study revealed that Gtf B enzyme activity was reduced by emodin, suggesting that emodin inhibits the synthesis of insoluble glucans partly through the suppression of Gtf B activity.

Acid production is an important dental caries-related factor of *S. mutans* (28–30). In dental biofilms, *S. mutans* metabolizes sugars and produces organic acids including lactic, propionic, formic and butyric acids. A concentration of organic acids may demineralize the tooth surface and thereby induce dental caries (2,3). In the present study, emodin significantly reduced the level of acid produced by *S. mutans*. This inhibitory activity of emodin may be due to its effect on the bacterial membrane. Emodin has a high affinity for phospholipid membranes and is able to disrupt the hydrophobic interactions between hydrocarbon chains in phospholipid bilayers (31). The inhibitory effect of emodin on the production of acid by *S. mutans* may occur through the disruption of the bacterial cell membrane and, thus, the inhibition of the expression levels and activities of specific proteins associated with sugar transport and metabolism.

The inhibitory effect of emodin on the growth, insoluble glucan synthesis and acid production of *S. mutans* may be beneficial for the prevention of the formation of cariogenic biofilms *in vivo*. Therefore, the present study further examined the anti-caries activity of emodin using a rat model of dental caries. The topical application of emodin reduced the incidence and severity of carious lesions in rats without affecting the percentage of *S. mutans* in the biofilms. In smooth-surface caries, emodin effectively reduced the abundance and severity of the caries, which was similar to that of the positive control (250 ppm fluoride). However, the inhibitory effect of emodin on sulcal caries was not as effective as the 250 ppm fluoride control treatment. These results suggest that the anti-caries mechanism of emodin may be attributed to multiple inhibitory effects, including inhibition of the growth, insoluble glucan and acid production of *S. mutans*. Furthermore, matrix metalloproteinases are involved in the pathogenesis of dental caries (32). Tetracycline-3, an inhibitor of matrix metalloproteinases, is effective in the prevention of dental caries (33). Emodin has been demonstrated to inhibit the activity and expression of matrix metalloproteinases *in vitro* and *in vivo* (34,35). Thus, the anti-caries activity of emodin may also be due to its inhibitory effect on matrix metalloproteinases. Further study is required to investigate this issue.

In summary, the results of the present study revealed that emodin significantly attenuated the growth, acid production and insoluble glucan synthesis of *S. mutans in vitro*, and suppressed the development of dental caries in rats. These results suggest that emodin may be a novel therapeutic agent for the prevention and control of dental caries.

## Figures and Tables

**Table I:** Effect of emodin on the oral microbiota of rats following a five-week experiment.

Group	Total microorganisms (×10^4^ cfu/ml)	*S. mutans* ATCC 25175 (×10^4^ cfu/ml)	*S. mutans* ATCC 25175 (%)
Vehicle	4.0^a^ (1.2)	2.7^a^ (1.8)	67.9^a^ (20.7)
2 mg/ml emodin	2.2^b^ (0.6)	1.4^a^ (0.4)	63.6^a^ (16.8)
250 ppm fluoride	2.3^b^ (0.5)	1.5^a^ (0.2)	65.2^a^ (13.5)

Data are presented as the mean with the standard error of the mean in parentheses following the statistical analyses of all pairs using the Tukey-Kramer multiple comparison test (n=15). Fluoride (250 ppm) was used as a positive control. Values followed by different superscript letters are significantly different from one other (P<0.05). *S. mutans, Streptococcus mutans*; cfu, colony-forming unit.

**Table II:** Effect of various treatments on smooth-surface caries development (incidence and severity) in rats.

		Severity		
		
Group	Total smooth surface	Ds	Dm	Dx
Vehicle	67.2^a^ (6.6)	40.8^a^ (7.8)	16.8^a^ (6.1)	6.2^a^ (6.4)
2 mg/ml emodin	42.6^b^ (5.8)	24.7^b^ (6.9)	5.4^b^ (7.1)	2.2^b^ (0.7)
250 ppm fluoride	22.7^c^ (2.5)	18.6^b^ (7.6)	0.9^b^ (0.5)	0.2^c^ (0.3)

Data are presented as the mean with the standard error of the mean in parentheses following the statistical analyses of all pairs using the Tukey-Kramer multiple comparison test (n=15). Keyes’ scores followed by different superscript letters are significantly different from one other (P<0.05). Ds, dentin exposed; Dm, 3/4 of the dentin affected; Dx, whole dentin affected.

**Table III:** Effect of various treatments on sulcal-surface caries development (incidence and severity) in rats.

		Severity		
		
Group	Total sulcal surface	Ds	Dm	Dx
Vehicle	36.3^a^ (5.3)	27.2^a^ (4.2)	20.8^a^ (4.3)	15.4^a^ (6.2)
2 mg/ml emodin	30.4^b^ (4.2)	18.1^b^ (3.2)	10.1^b^ (4.2)	6.3^b^ (3.8)
250 ppm fluoride	19.3^c^ (2.3)	10.2^c^ (3.5)	3.2^c^ (1.6)	0.7^c^ (0.6)

Data are presented as the mean with the standard error of the mean in parentheses following the statistical analyses of all pairs using the Tukey-Kramer multiple comparison test (n=15). Keyes’ scores followed by different superscript letters are significantly different from one other (P<0.05). Ds, dentin exposed; Dm, 3/4 of the dentin affected; Dx, whole dentin affected.

## References

[1] Loesche WJ (1986). Role of *Streptococcus mutans* in human dental decay. Microbiol Rev.

[2] Bowden GH (1990). Microbiology of root surface caries in humans. J Dent Res.

[3] Belli WA, Marquis RE (1991). Adaptation of *Streptococcus mutans* and *Enterococcus hirae* to acid stress in continuous culture. Appl Environ Microbiol.

[4] Yamashita Y, Bowen WH, Burne RA, Kuramitsu HK (1993). Role of the *Streptococcus mutans* gtf genes in caries induction in the specific-pathogen-free rat model. Infect Immun.

[5] Bowen WH (2002). Do we need to be concerned about dental caries in the coming millennium?. Crit Rev Oral Biol Med.

[6] Branco-de-Almeida LS, Murata RM, Franco EM (2011). Effects of 7-epiclusianone on *Streptococcus mutans* and caries development in rats. Planta Med.

[7] Badria FA, Zidan OA (2004). Natural products for dental caries prevention. J Med Food.

[8] Zhou Z, Miwa M, Nara K (2003). Patch establishment and development of a clonal plant, *Polygonum cuspidatum*, on Mount Fuji. Mol Ecol.

[9] Park CS, Lee YC, Kim JD, Kim HM, Kim CH (2004). Inhibitory effects of *Polygonum cuspidatum* water extract (PCWE) and its component resveratrol [correction of rasveratrol] on acyl-coenzyme A-cholesterol acyltransferase activity for cholesteryl ester synthesis in HepG2 cells. Vascul Pharmacol.

[10] Song JH, Kim SK, Chang KW, Han SK, Yi HK, Jeon JG (2006). *In vitro* inhibitory effects of *Polygonum cuspidatum* on bacterial viability and virulence factors of *Streptococcus mutans* and *Streptococcus sobrinus*. Arch Oral Biol.

[11] Ban SH, Kwon YR, Pandit S, Lee YS, Yi HK, Jeon JG (2010). Effects of a bio-assay guided fraction from *Polygonum cuspidatum* root on the viability, acid production and glucosyltranferase of mutans streptococci. Fitoterapia.

[12] Chang CH, Lin CC, Yang JJ, Namba T, Hattori M (1996). Anti-inflammatory effects of emodin from *Ventilago leiocarpa*. Am J Chin Med.

[13] Zhou M, Xu H, Pan L, Wen J, Guo Y, Chen K (2008). Emodin promotes atherosclerotic plaque stability in fat-fed apolipoprotein E-deficient mice. Tohoku J Exp Med.

[14] Tzeng TF, Lu HJ, Liou SS, Chang CJ, Liu IM (2012). Emodin protects against high-fat diet-induced obesity via regulation of AMP-activated protein kinase pathways in white adipose tissue. Planta Med.

[15] Matsumoto M, Minami T, Sasaki H, Sobue S, Hamada S, Ooshima T (1999). Inhibitory effects of oolong tea extract on caries-inducing properties of mutans streptococci. Caries Res.

[16] Choi BK, Kim KY, Yoo YJ, Oh SJ, Choi JH, Kim CY (2001). *In vitro* antimicrobial activity of a chitooligosaccharide mixture against *Actinobacillus actinomycetemcomitans* and *Streptococcus mutans*. Int J Antimicrob Agents.

[17] Venkitaraman AR, Vacca-Smith AM, Kopec LK, Bowen WH (1995). Characterization of glucosyltransferaseB, GtfC, and GtfD in solution and on the surface of hydroxyapatite. J Dent Res.

[18] Germaine GR, Schachtele CF, Chludzinski AM (1974). Rapid filter paper assay for the dextransucrase activity from *Streptococcus mutans*. J Dent Res.

[19] Bowen WH, Madison KM, Pearson SK (1988). Influence of desalivation in rats on incidence of caries in intact cagemates. J Dent Res.

[20] Koo H, Pearson SK, Scott-Anne K (2002). Effects of apigenin and tt-farnesol on glucosyltransferase activity, biofilm viability and caries development in rats. Oral Microbiol Immunol.

[21] Gazzani G, Daglia M, Papetti A (2012). Food components with anticaries activity. Curr Opin Biotechnol.

[22] Koo H, Schobel B, Scott-Anne K (2005). Apigenin and tt-farnesol with fluoride effects on *S. mutans* biofilms and dental caries. J Dent Res.

[23] Kim JE, Kim HJ, Pandit S, Chang KW, Jeon JG (2011). Inhibitory effect of a bioactivity-guided fraction from *Rheum undulatum* on the acid production of *Streptococcus mutans* biofilms at sub-MIC levels. Fitoterapia.

[24] Hotz P, Guggenheim B, Schmid R (1972). Carbohydrates in pooled dental plaque. Caries Res.

[25] Schilling KM, Bowen WH (1992). Glucans synthesized *in situ* in experimental salivary pellicle function as specific binding sites for *Streptococcus mutans*. Infect Immun.

[26] Madison KM, Bowen WH, Pearson SK, Falany JL (1991). Enhancing the virulence of *Streptococcus sobrinus* in rats. J Dent Res.

[27] Koo H, Xiao J, Klein MI, Jeon JG (2010). Exopolysaccharides produced by *Streptococcus mutans* glucosyltransferases modulate the establishment of microcolonies within multispecies biofilms. J Bacteriol.

[28] Harper DS, Loesche WJ (1984). Growth and acid tolerance of human dental plaque bacteria. Arch Oral Biol.

[29] Bender GR, Thibodeau EA, Marquis RE (1985). Reduction of acidurance of streptococcal growth and glycolysis by fluoride and gramicidin. J Dent Res.

[30] Kuramitsu HK (1993). Virulence factors of mutans streptococci: role of molecular genetics. Crit Rev Oral Biol Med.

[31] Alves DS, Pérez-Fons L, Estepa A, Micol V (2004). Membrane-related effects underlying the biological activity of the anthraquinones emodin and barbaloin. Biochem Pharmacol.

[32] Chaussain-Miller C, Fioretti F, Goldberg M, Menashi S (2006). The role of matrix metalloproteinases (MMPs) in human caries. J Dent Res.

[33] Sulkala M, Wahlgren J, Larmas M (2001). The effects of MMP inhibitors on human salivary MMP activity and caries progression in rats. J Dent Res.

[34] Lee J, Jung E, Lee J, Huh S (2006). Emodin inhibits TNF α-induced MMP-1 expression through suppression of activator protein-1 (AP-1). Life Sci.

[35] Wierzchacz C, Su E, Kolander J, Gebhardt R (2009). Differential inhibition of matrix metalloproteinases-2, -9, and -13 activities by selected anthraquinones. Planta Med.

